# Analytical Determination of Heavy Metals in Human Seminal Plasma—A Systematic Review

**DOI:** 10.3390/life13040925

**Published:** 2023-03-31

**Authors:** Andrea López-Botella, Raquel Sánchez, Raiza Paul, Jon Aizpurua, María José Gómez-Torres, José-Luis Todolí-Torró

**Affiliations:** 1 Biotechnology Department, Faculty of Sciences, University of Alicante, Carretera San Vicente del Raspeig s/n, 03690 Alicante, Spain; andrea.lopez@ua.es (A.L.-B.); mjose.gomez@ua.es (M.J.G.-T.); 2 Department of Analytical Chemistry, Nutrition and Food Sciences, University of Alicante, Carretera San Vicente del Raspeig s/n, 03690 Alicante, Spain; r.sanchez@ua.es; 3 iGLS, Av. de Ansaldo, 12, 03540 Alicante, Spain; r.paul@igls.net; 4 IVF Spain, Reproductive Medicine, 03540 Alicante, Spain; j.aizpurua@ivf-spain.com

**Keywords:** heavy metals, seminal plasma, environmental factors, lifestyle factors, seminal biomarkers, male infertility, endocrine-disrupting compounds

## Abstract

Infertility is a growing concerning health problem affecting around 15% of couples worldwide. Conventional semen parameters have limited accuracy for male infertility potential determination. Current advances in the understanding of male infertility indicate that environmental and occupational exposure to chemical contaminants are important etiological factors leading to infertility problems. In this context, some heavy metals (HMs) can be considered as endocrine-disrupting compounds (EDCs), thus altering the seminal quality. This systematic review aims to summarize the key points to detect and quantify HMs in human seminal plasma (SP) and the involved analytical tools. Our results showed that that for HM quantification, atomic absorption spectroscopy (AAS) and inductively coupled plasma (ICP) were the most employed techniques while Zn, Cd, Pb, and Cr were the analytes most often detected. Fast, reliable, and sensitive quantification of EDCs in SP could be important for the development of accurate diagnostic and preventive strategies to address male infertility towards providing personalized therapy.

## 1. Introduction

Infertility is a major concerning health problem affecting around 15% of couples worldwide [1]. Specifically, male partners are responsible for 20–30% of the overall infertility cases [2], although this percentage depends on the geographical region and country. The decline in male fertility is evidenced from available studies suggesting lower semen quality over the years [3], thus being related to infertility problems. Male infertility can be caused by several factors appearing in isolation or association with various complex syndromes [4].

Current advances in the understanding of male infertility indicate that environmental [5,6] and occupational exposure [7] to chemical contaminants are important etiological factors leading to infertility problems [5]. In this context, some heavy metals are considered endocrine-disrupting compounds, which are environmental and occupational contaminants [5,7]. The term “heavy metals” refers to “naturally occurring metals having an atomic number (Z) greater than 20 and an elemental density greater than 5 g cm^−3^” [8]. However, in the case of environmental studies, the term “potentially toxic element(s)” (PTEs) has been proposed as it is a wider and less ambiguous term [9]. We will use the term “heavy metals” as we have previously used it in a recent review [10]. Chemical elements, such as arsenic (As), cadmium (Cd), lead (Pb), and mercury (Hg) have been included within the EDC category [11], as they are exogenous species involved in processes regulated by endogenous hormones altering the normal functioning of the endocrine system in humans [12]. The results from a review recently published by our research group revealed that men exposed to heavy metals had a lower seminal quality, which was related to infertility or to a subfertility status. Our bibliographical findings suggested that Pb and Cd are the analytes that most negatively affect seminal quality, being correlated with a lower sperm concentration, an abnormal spermatic morphology, and lower sperm viability [10]. Nevertheless, human seminal fluid contains trace elements such as calcium (Ca), copper (Cu), manganese (Mn), magnesium (Mg), zinc (Zn), and selenium (Se), which are crucial for reproductive health and normal sperm function. In fact, Zn and Se are the two main elements with antioxidant properties [13].

The identification of pollutants of interest in seminal fluid could help to clarify their effect on male reproductive function [7]. However, the sensitivity and specificity of seminal fluid concentration as a marker of occupational (or non-occupational) exposure has only been tested for a few substances, with diverse results [7]. So, whether the presence of HMs in seminal plasma (SP) and semen highlights environmental and occupational exposure remains unknown. Although the concentration of contaminants in seminal fluid is lower than in the blood and urine [7] and generally, the evidence relies on urinary concentrations, EDC quantification in seminal plasma might provide more information about the potential toxicity that could arise in the testes [14].

Conventional semen parameters have limited accuracy for the potential determination of male infertility, since 30–40% of cases involve unexplained or idiopathic male infertility [1]. Additionally, it should be noted that basic semen analysis is not the most suitable analysis to obtain an accurate diagnosis because semen parameters within the reference interval do not guarantee fertility. It has been estimated that 15% of infertile men present normal sperm parameters. This value requires additional tests to investigate the causes of infertility and accurately determine the factors altering sperm quality [4]. In addition, routine sperm analysis provides little qualitative and functional information about sperm cells together with their fertilizing ability. Due to this, new functional tests are needed to develop and provide newer biomarkers of sperm’s fertilizing capacity [15]. The physiological and functional assessment of sperm should also include molecular biomarkers such as reactive oxygen species (ROS), DNA damage, and chromatin structure [16].

Genetic biomarkers of semen quality are advantageous as they can indicate toxic effects derived from environmental pollution and describe male reproductive capacity [17]. DNA damage caused by oxidative processes induced by free oxygen radicals [18], which are derived from xenobiotics, had been considered a molecular key mechanism associated with semen quality and sperm functionality [17]. Recently, it has been reported that occupationally exposed infertile men (tea garden workers of Assam, India) had high levels of Pb and Cd in their seminal plasma, together with anomalous oxidative stress and sperm parameters [19]. In this research, the group of men with higher concentrations of Pb and Cd had higher percentages of DNA-damaged cells.

Recently, the literature has pointed out environmental exposure to EDCs and lifestyle (stress and diet) as factors that could alter the epigenetic mechanisms in the spermatozoa, because cellular epigenetics are very plastic and can be easily influenced by the environment [20]. Limited information is available about how heavy metals induce epigenetic changes. In human subjects, Pb was found to compete or replace the naturally present Zn in human P2 (protamine P2) in vivo and hence, it could alter chromatin condensation [21]. For their part, Cd and hexavalent chromium (Cr (VI)) may alter the epigenetic mechanisms in mice spermatozoa. Cd has been shown to induce massive and aberrant lncRNA and mRNA expression profiles in testes and spermatozoa, being related to a lower testicular sperm production, motility, and normal morphology [22], while Cr (VI) seems to produce an increase in H3K9me3 and H3K27me3 as well as the activation of the apoptotic signaling pathway in spermatogonial stem cells [23].

The description of the molecular profile and phenotypes of infertility resulting from the interaction between genetic and environmental factors is a key point for screening the greatest informative biomarkers, assessing their potential, and validating new molecules as potential targets for personalized therapy [24]. Therefore, this systematic review reports basic and clinical studies using novel approaches in heavy metal detection and quantification in seminal plasma in order to offer personalized medicine and preventive strategies addressing male infertility.

## 2. Materials and Methods

### 2.1. Search Strategy

#### Heavy Metals in Seminal Plasma

As in previous reviews published by our research group [10,25], in this systematic review, the same steps were followed. First, generic searches were performed using the Google Scholar portal (https://scholar.google.es/, accessed on 20 November 2021). This allowed us to identify key concepts about how heavy metals are quantified in seminal plasma, but also aided in selecting the final keywords to use in a more comprehensive search through some specific scientific databases. The ultimate list of keywords derived from the Medical Subject Heading (MeSH) database and National Library of Medicine (NLM). Thus, resulted in selecting the keywords “Seminal Plasma” and “Heavy Metals”. We noticed that some articles used the word “semen” to refer to seminal plasma, so we decided to choose it as a keyword.

A full search was performed in online databases related to the issue under study to achieve an accurate bibliometric and bibliographical analysis and to be aware of the bibliographic load indexed in each one of the online databases. We selected Scopus, PubMed, and Web of Science (WOS) as databases. The search was performed, as previously described, using the “Boolean” system which allowed us to identify the studies of interest to construct this review using the following combinations of keywords: “Seminal Plasma and Heavy Metals” and “Semen and Heavy Metals” in each of the databases (accessed from 4 November 2021 to 14 April 2022).

The “Search all databases” option was selected during this research to ensure items were not lost, and the information retrieval system of the “Boolean” model helped to identify the research of interest for this review by using the keyword combinations previously mentioned. An attached database was created in Excel to include the articles obtained from each single search result. The search and selection of relevant studies was performed by the first author and every step was supervised by the corresponding author.

### 2.2. Selection of Relevant Studies and Data Analysis

#### Heavy Metals in Seminal Plasma

**Figure 1:**
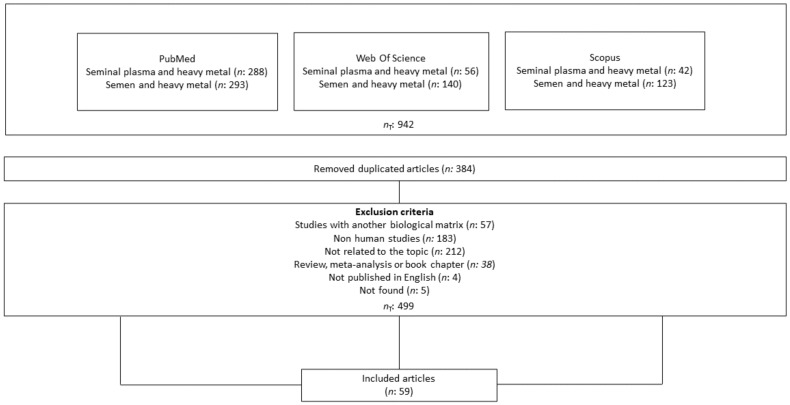
Flowchart summarizing the selection process of the articles on heavy metal determination included after the inclusion criteria application. Adapted from the PRISMA Group [26].

We incorporated two inclusion criteria which helped to filter the results and served to select the documents: (i) from 2010 to 2021 and (ii) articles from primary sources and indexed journals. To construct this systematic review, we selected the following exclusion criteria: (i) studies with a different biological matrix than seminal plasma or semen; (ii) non-human studies; (iii) studies not related to the topic; (iv) reviews, meta-analysis studies, or book chapters; (v) studies not published in English; and (vi) not found articles. The flowchart in Figure 1 summarizes the search and the steps followed until the selection of the articles analyzing HMs in SP that were finally included in this review.

## 3. Results and Discussion

### 3.1. Compilation of Relevant Bibliographical Sources

A total of 942 articles was obtained from the PubMed, Web of Science, and Scopus databases after the inclusion criteria application. The elimination of duplicate articles (*n*: 384) and the removal of those that met the exclusion criteria (*n*: 499) enabled us to build the final database. The final number of articles included in this review was 59, approximately 6% of the total articles initially found.

### 3.2. Bibliometric Analysis

**Figure 2:**
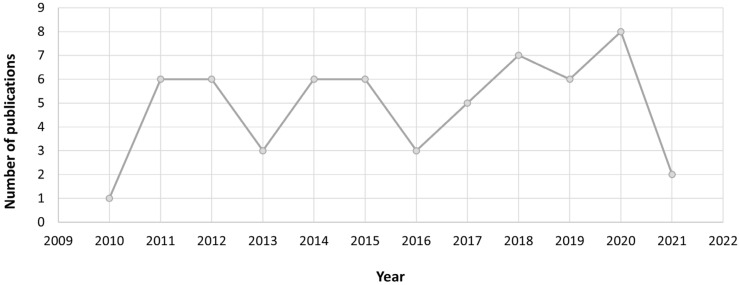
The number of publications by year (January 2010–December 2021), including all the database articles created after the application of inclusion and exclusion criteria.

The keyword search showed that the number of publications did not follow a clear trend. The analysis of the 59 articles revealed that while in specific years (2013, 2016, and 2021), the number of publications decreased, it reached its highest values in 2018 and 2020 with seven and eight studies, respectively (Figure 2). An increase in the number of publications could be noticed since 2016, with particularly 53% of the total number of articles having been published since that year. These findings could be explained by emerging worries about environmental exposure to heavy metals and its relationship with human male infertility [10].

Focusing on the journals that published the articles about our topic under study, Biological Trace Element Research (*n*:7) together with Andrologia and Environmental Science and Pollution Research (*n*:3) were the journals with the highest number of publications. India and China were principally the countries to which the first authors of the publications belonged (Table 1).

### 3.3. Bibliographical Analysis

#### 3.3.1. Heavy Metal Detection and Determination in Human Seminal Plasma

**Figure 3:**
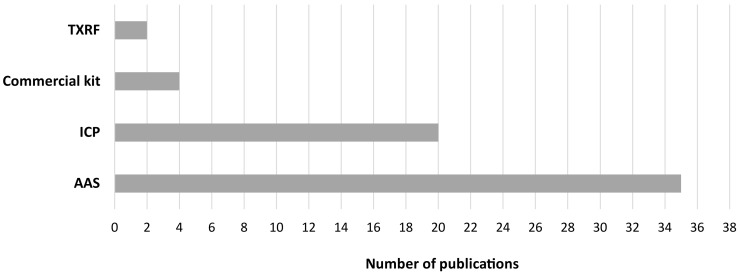
Analytical techniques employed to perform heavy metal determination in seminal plasma.

The analytical techniques employed in our 59 included articles were atomic absorption spectroscopy (AAS), inductively coupled plasma (ICP), total reflection X-ray fluorescence (TXRF), and commercial kits. Two of the investigated articles from the total of 59 employed two analytical techniques (Figure 3), which were TXRF together with inductively coupled plasma optical emission spectroscopy (ICP-OES) and mass spectrometry (ICP-MS) with AAS. In short, the most employed analytical techniques for heavy metal detection and quantification in seminal plasma were AAS and ICP (35 and 20 published articles, respectively).

In this systematic review, a summary of the technical characteristics used in each article will be conducted, according to the employed analytical technique. A list of the heavy metals detected and/or quantified in seminal plasma as well as the sample preparation for the analysis will be included. A table with the chemical elements and their symbols is available to help with the understanding of this article (Table 2).

#### 3.3.2. Atomic Absorption Spectroscopy

An important issue to be considered when AAS is to be applied is related to the accuracy of determinations. In fact, the so-called interferences may negatively impact the reliability of the obtained concentrations and give rise to false results. In other words, the matrix composition significantly affects the analytical results. Therefore, the sample composition should be considered, together with the impact of each one of the components, on the measured absorbance.

Citrate is one of the most abundant anions in seminal plasma [51]. This anion has an affinity to elements such as Ca, Mg, and Zn, among others, and can also act as a reducing agent for several ions (e.g., Hg(II) to Hg(0) [52]). Furthermore, citrate is used as a chemical modifier in graphite furnace AAS (GFAAS) [53]. Indeed, its presence can modify the stability of elements during the pyrolysis step and lead to inaccurate results if the compound is not properly added to the standards. Moreover, within the category of organic compounds, glucose and fructose are significant sources of energy for sperm and are present at levels of hundreds of milligrams per 100 mL. These compounds yield spectral as well as non-spectral interferences [54]. Together with these concomitants, proteins are present at an average concentration close to 5000 mg/100 mL which adds more complexity to the analysis by means of atomic absorption techniques.

As expected, elements such as Ca, Mg, K, Na, and Zn are present at relatively high concentrations in seminal plasma samples [51]. All these elements may cause a modification in the instrument’s performance because most of them are easy to ionize and, hence, lead to a shift in the analyte ionization equilibrium in the flame towards the atomic state. As a result, absorbance increases in the presence of these elements, thus giving rise to an overestimation of the elemental content [55]. As a result of all these drawbacks, samples must be prepared (conditioned) before their introduction into the spectrometer. The most common methods are based on either sample dilution or its digestion to decompose organic matter. These methods will be mentioned in the following sections.

##### Sample Preparation for AAS Determination

It is important to avoid the contamination of the samples, so to prevent this the glassware [32] or the polyethylene tubes [49] can be immersed in nitric acid. Then, the instrumental was passed through water, distilled water, deionized water, distilled, and redistilled deionized water before use. For seminal plasma preparation and AAS determination, firstly seminal plasma was separated by centrifuging the semen at 300× *g* for 7 min [48], 1500× *g* [27] or 600× *g* for 10 min [49], 612.3× *g* for 30 min [29], and was even recentrifuged at 100,000× *g* for two hours [49]. A step of deproteinization by adding tricarboxylic acid before centrifugation also took place in one study [56]. Then, the seminal plasma may be transferred to a clean, dry plain container and stored at −20 [57], −40 [58], or −80 °C [59] until its analysis. Zn, for instance, could be measured directly by flame AAS [60] after sample dilution with deionized water [61]. Other elements, such as Cu, Fe, and Se were measured after dilution by GFAAS [61].

Acid digestion is a usual method for sample preparation to ensure the removal of organic impurities and prevent interferences. Digestion with concentrated nitric acid [3,62] alone or together with hydrogen peroxide (H_2_O_2_) in a Teflon digestion vessel placed in a closed microwave system [57,63,64] or using with perchloric acid [19,29,48,61] instead of H_2_O_2_ can be conducted. The wet digestion method releases metals from the protein matrix by adding nitric and hydrochloric acid [65]. After digestion, the sample is filtered and diluted using deionized water [57,63,64] or can be dissolved in nitric acid [29] before the determination.

##### Instrumental Settings for Heavy Metal Detection and Quantification

Out of our final database search, 35 articles used AAS for heavy metal detection and quantification in human seminal plasma. Among the spectroscopy techniques, Flame atomic absorption spectroscopy (FAAS) and GFAAS were the most widely used (Table 3). Articles were examined to find specifications about the atomic absorption spectroscopy equipment (Table S1). Thus, the information about the limits of detection, recoveries, conditions for measuring elements, and calibration and quality control information were collected. A total of 34% of the studies based on AAS for heavy metal determination provided values for the limits of detection (Table 3), while only three articles (9%) had recovery information. Interestingly, 45% of the articles included information about calibration techniques and quality control.

##### Heavy Metal Detection and Quantification

**Figure 4:**
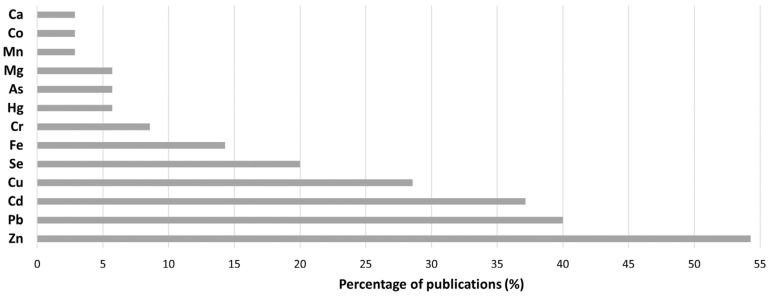
Detected and quantified analytes by AAS.

The most quantified analytes by AAS were Zn (54%), Pb (40%), Cd (37%), and Cu (29%) as shown in Figure 4. Cu, Se, and Fe were quantified by AAS in a great number of articles too. However, important heavy metals that also have a negative impact on male fertility such as Hg and As analytes were minimally measured by AAS.

As said previously, Zn, Pb, and Cd were the most quantified analytes by AAS. Because of this finding, the information about the quantification of these analytes in seminal plasma is summarized in Table 4 together with the study groups employed in each article.

Despite the limitations of AAS techniques, they have been successfully applied to the quantification of elements in SP, thus giving rise to useful clinical information. Among the included studies in this systematic review related to quantifying heavy metals in seminal plasma by AAS, different study groups of men were found. Often, they were divided by the following criteria: fertile men as the control group and non-fertile men [43,63,66]; normozoospermic (N) men as the control group and oligospermic (O) men [27,32,59,61,65,70,71,74]; azospermic (Azo) men [32,59,65,71,77]; asthenozoospermic (A) men [32,56,60,61]; teratozoospermic (T) men [61,70]; oligoasthenospermic (OA) [68,75,77]; and oligoasthenoteratospermic (OAT) men [48,58,60,68].

Fertile men, compared to infertile men, had lower concentrations of Cd and Pb in their SP [43,66] and higher amounts of Zn [43]. However, controversial results were found in the PB and Cd SP content in men diagnosed with O, Azo, A, T, OA, and OAT men compared to N men. In terms of Zn content, slightly or not at all different concentrations were found. Two articles showed a higher content of SP Zn in N men compared to A and O [70] men, or compared to T, AT and OT men [61].

Lifestyle habits were also tested [29,47]. Thus, for instance, in infertile smokers, the amount of Pb was higher compared to the control group while lower Zn levels were found in the former [29]. Further to this, Zn levels were found to be greater in fertile non-smokers compared to fertile smokers [47]. Likewise, environmental [57] and occupational exposure [19,57] was also investigated. In occupationally exposed men, higher levels of Cd were found in seminal plasma while Pb was found at higher levels in environmentally and occupationally exposed men [57].

Furthermore, differences in heavy metal content were found in groups of men after in vitro treatment where embryo transfer (ET) was achieved [76]. Thus, in the case of Zn, its levels were higher in the group of men who achieved ET. Further to this, the impact of metal implants on seminal plasma was also tested as one article studied the Cr and Co seminal plasma content in men with metal-on-metal and metal-on-polyethylene implants [72].

##### Limitations of Atomic Absorption Techniques

Although AAS techniques can be considered suitable for the determination of a given set of elements, they suffer from serious drawbacks which have led to the development of additional methods to perform the multi-elemental analysis of SP samples. Among the developed analytical tools, we can highlight inductively coupled plasma techniques, including optical emission (ICP-OES). This technique is also called ICP atomic emission spectroscopy (ICP-AES). However, according to the IUPAC criterion, the first acronym is more appropriate [78]. Included within this group of methods, inductively coupled plasma mass spectrometry (ICP-MS) is considered an excellent technique for trace multi-elemental analysis. Both techniques have emerged as alternatives to overcome the problems found when using AAS.

**Figure 5:**
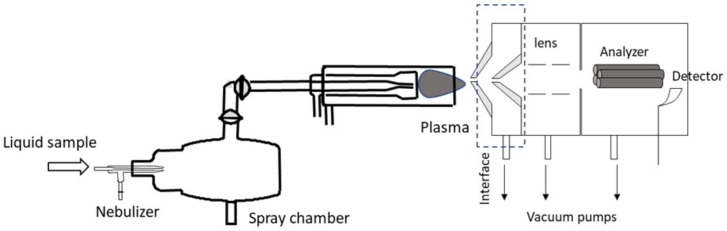
Main components of an ICP-MS instrument together (adapted from [80]).

A schematic of the major components of the ICP-MS instrument is available in Figure 5. First, the sample is usually introduced into the plasma as a liquid solution that is first transformed into an aerosol by means of a nebulizer. Because nebulizers are not able to generate fine enough aerosols, a second component of the sample introduction system (i.e., the so-called spray chamber) should be used. Its main purpose is to select the finest aerosol droplets. Once these small droplets (generally having diameters below 20 μm) reach the plasma, they are evaporated and the analyte they contain is atomized, ionized, and/or excited, depending on the ICP technique. Note that ICP is a plasma source containing free atoms, ions, and electrons and whose temperature ranges from roughly 4000 to 10,000 K [55]. Therefore, it has an amount of energy high enough to ionize the free atoms of most of the elements listed in Table 2. In many cases, free ions can even be excited. It is worth mentioning that the quality of the analytical results finally obtained depends strongly on the characteristics of the sample introduction system. Therefore, many different configurations have been developed and tested [79].

**Figure 6:**
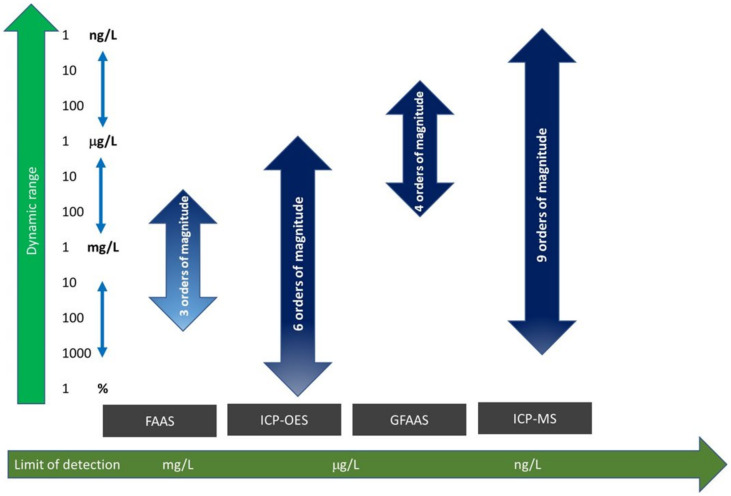
Comparison of the different spectrometric techniques in terms of the linear dynamic range (y-axis) and limit of detection (x-axis) (adapted from [80]).

A comparison of the different techniques employed for the analysis of SP samples is presented in Figure 6. The criteria established in this figure are the limits of detection (x-axis) and dynamic linear range (y-axis) that correspond to the concentration range for which there is a linear relationship between the analytical signal and the analyte concentration. According to this figure, it emerged that ICP-MS followed by ICP-OES are the best techniques for trace analysis. First, the limits of detection provided by ICP-MS are the lowest (Table 5) among the techniques considered and, second, the dynamic range is wider for both ICP methods than for AAS ones. The latter point is highly relevant, because it implies that ICP-OES and ICP-MS allow for the analysis of samples with very low as well as high analytical concentrations without the need for a previous sample dilution, thus enormously simplifying the analysis method. It should also be mentioned that unlike in AAS, they are able to determine around 40–50 different elements in the same analytical run. Despite their higher cost and the fact that they also suffer from interferences, ICP techniques have a wide acceptance for use in the multi-elemental analysis of clinical samples.

#### 3.3.3. Inductively Coupled Plasma (ICP)

For heavy metal detection and quantification in human seminal plasma by ICP, a total of 20 articles was included in our final database search results. Among the spectrometric techniques, the most widely used was ICP-MS (*n*: 15) followed by ICP-OES/AES (*n*: 5) (Table 5).

As previously mentioned for AAS techniques, the sample matrix may have a direct impact on the instrument performance. Within the ICP field, several studies describing in detail the different sources of interference have been published. According to the SP composition, two main groups of compounds can be distinguished: organic species and dissolved salts.

The introduction of organic species in ICP techniques may have several risks [92]: (i) The presence of carbon causes a soot build-up either in the torch or the ICP-MS interface. This leads to a signal drift and sensitivity degradation. To overcome this problem, oxygen can be added prior to the plasma and organic matter will decompose, generating CO_2_. (ii) The degradation of the plasma’s thermal state can be observed because a given amount of energy is taken from the plasma to dissociate the different matrix components. To avoid this problem, the plasma is usually operated under so-called robust conditions (i.e., high plasma RF power and low nebulizer gas flow rate). (iii) Charge transfer reactions are produced between carbon ions originating in the plasma’s central channel and the analyte. This process yields an enhancement in ionic signals and, to mitigate it, carbon should be added to the standards. Compounds such as those present in SP (e.g., citrates, sugars, and proteins) may induce any of the aforementioned phenomena [93].

Apart from these processes, spectral interferences when organic species are present in ICP techniques are especially relevant [94]. The nature of spectral interferences depends on the particular technique used. Thus, in the case of ICP-OES, they are referred to as the overlap between the analytical emission peaks and emission bands caused by carbon byproducts. Among them, we could highlight carbon light-emitting atoms and ions, carbon monoxide-excited molecules, CN, and carbon dimers (C_2_). Elements such as Ni or Co, among others, can be affected by these interferences.

When working with ICP-MS, spectral interferences originate where an ion generated from the sample matrix (mainly a polyatomic ion) has a similar mass-to-charge ratio as that for the analyte nuclide. In order to illustrate the complexity of this situation, a list of possible spectral interferences is given in Table 6. Besides these interferences, it should be mentioned that polyatomic ions are also generated within the plasma even in presence of plain water solutions (e.g., ArO^+^, Ar_2_^+^). These ions make the determination of important elements such as Fe and Se fairly difficult.

As mentioned before, inorganic dissolved salts are also present in the SP matrix. Thus, it has been widely indicated that elements such as Na, K, and Ca, among others, cause severe interferences both in ICP-OES and ICP-MS. In fact, the so-called easily ionized elements may lead to a change in the processes taking place inside the ICP that leads to a degradation in the accuracy of the results. Moreover, in ICP-OES, the extent and nature of the interferences depends on the plasma observation zone [95]. Thus, if the signal is taken from the plasma base, the presence of this kind of element causes a decrease in analytical sensitivity as compared to a plain water solution. In contrast, when taking the signal up in the plasma, easily ionized elements induce an enhancement of the sensitivity which leads to an overestimation of the analyte concentration [96]. In order to prevent these so-called non-spectroscopic interferences (or matrix effects), several methodologies can be applied. Among them, internal standardization is the most often used [83]. Thus, a series of elements is added to the sample either inline or manually. Then the signals found for the analytes are ratioed with respect to those measured for the elements used as internal standards. A good internal standard candidate must not be present in the original sample and be exposed to the same interferences as the analyte of interest. A generally accepted criterion in ICP-MS is that the element to be used as the internal standard must have a mass-to-charge ratio as close as possible to that of the analyte. This is widely accepted, although, for some common matrices such as nitric acid, the first ionization potential of the chemical element appears to be the key property [97]. This may explain why, to perform multi-elemental seminal plasma analyses, several elements are simultaneously added to the samples as internal standards [37,83]. Common internal standards for ICP-MS are lithium (^6^Li), scandium (^45^Sc), germanium (^72^Ge), yttrium (^89^Y), rhodium (^103^Rh), indium (^115^In), tellurium (^125^Te), terbium (^159^Tb), rhenium (^185^Re), and iridium (^191^Ir). The use of Sc, Ge, Rh, Tb, or Lu has been reported for the analysis of seminal plasma samples [98].

As mentioned for organic concomitants, when working with ICP-MS, besides the phenomena occurring in ICP-OES, spectral interferences are still induced by dissolved salts. Thus, for instance, the presence of chloride ions may lead to peak overlapping between polyatomic ions and several analytes (Table 6).

In order to remove ICP-MS spectral interferences, several approaches have been developed [99], including the following : (i) monitorization of an isotope free of interference; (ii) modification of the sample introduction system; (iii) modification of the plasma operating conditions; (iv) use of a collision–reaction cell placed after the mass analyzer; (v) use of high-resolution mass spectrometry; or, more recently, (vi) use of ICP tandem mass spectrometry (ICP-MS/MS) [100].

**Figure 7:**
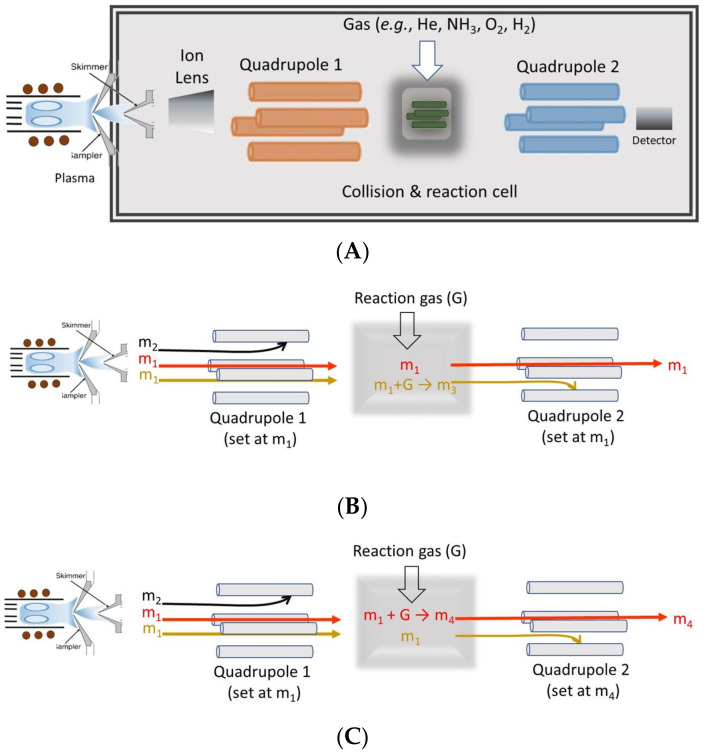

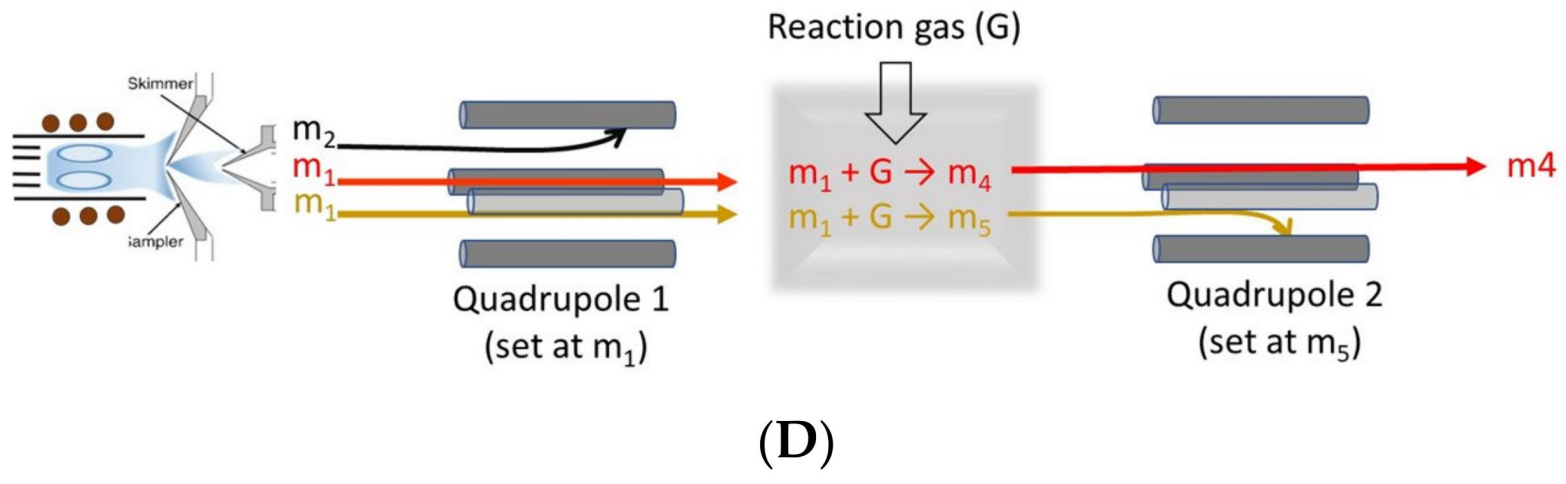
(**A**) Schematic of an ICP-MS/MS device and (**B**) strategies followed to overcome spectral interferences (m1, red, analyte; m1, yellowish, interferent). (**C**) On-mass approach and (**D**) mass-shift approach. (Adapted from [80]).

The latter technique is expected to be of increasing use because of the excellent analytical performance and robustness it shows with samples containing organic matter and salts. In this case, the configuration of the mass spectrometer is different as compared to conventional devices. An octopole cell is placed after the first quadrupole analyzer. Therefore, interfering ions leave it to interact with either a reaction gas (e.g., oxygen, hydrogen, ammonia, fluoromethane) or collision gas (e.g., helium). As a result, the interfering species are transformed into ions whose mass-to-charge ratios differ from that of the analyte. Alternatively, analyte ions react with the gas thus yielding adducts with a mass-to-charge ratio higher than that of the interfering ions. Once the ion beam leaves the octopole, it is driven to a second quadrupole analyzer whose main purpose is to separate ions containing the analyte from the remaining ones. This is achieved by tuning the second quadrupole to the appropriate mass-to-charge ratio. Figure 7 shows a schematic of an ICP-MS/MS together with the three strategies that can be applied in order to overcome spectral interferences.

##### Sample Preparation for Determination

After semen collection, the liquefied sample must be centrifuged for the seminal plasma preparation [86]. A simple step of dilution can be performed by adding ultra-pure water before measuring by ICP-OES [90]. However, sample dilution has two main problems: first, the obvious reduction in the analyte concentration that must be determined and, second, the fact that, although diluted, the matrix may induce a residual interference, thus leading to either an under- or overestimation of the concentration of heavy metals.

Seminal fluid aliquots can be digested with HNO3 [38], then diluted [40,81,84,87] and directly measured by ICP. Prior to HNO3 digestion, an evaporation using a mineralizer can be performed [44,45]. A mixture of HNO3 and H2O2 [30,31,37,82] or HNO3 and perchloric acid [34] can be used to digest the samples, followed by an acid evaporation at 120 °C, a dilution, and a filtration to avoid the presence of solid particles in the sample that could lead to nebulizer blockage [37]. HCl can also be used for the sample digestion [47]. Acid digestion can be performed in a closed vessel microwave system [30,31,81,82,85,89] that limits the loss of volatile elements and sample contamination. Again, the use of sample digestion leads to a dilution of the sample. However, additional matrix effects caused by the acids used may remain if the acid concentration is not compensated in the standards.

**Figure 8:**
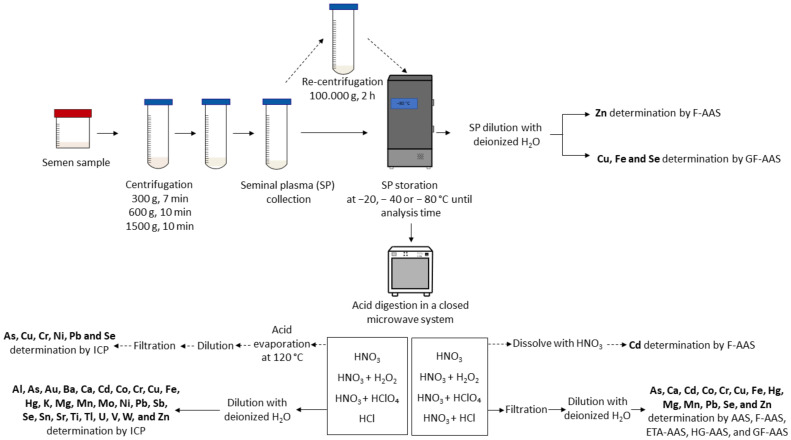
Sample preparation for HM detection in SP by AAS and ICP.

In short, as AAS and ICP are the most employed techniques to detect and quantify HMs in SP, a simple summary of the sample preparation before its assessment is available in Figure 8.

##### Instrumental Settings for Heavy Metal Detection and Quantification

Articles were studied to find information about the equipment specifications for the ICP technique (Table S1). Information about the limits of detection, recoveries, calibration, and quality control information were collected. An amount of 45% of the total articles that used ICP for heavy metal determination showed values for the limits of detection/quantification (Table 7), while only five articles (25%) had recovery information. Remarkably, 65% of the articles included information about calibration techniques and quality control.

##### Heavy Metal Detection and Quantification

**Figure 9:**
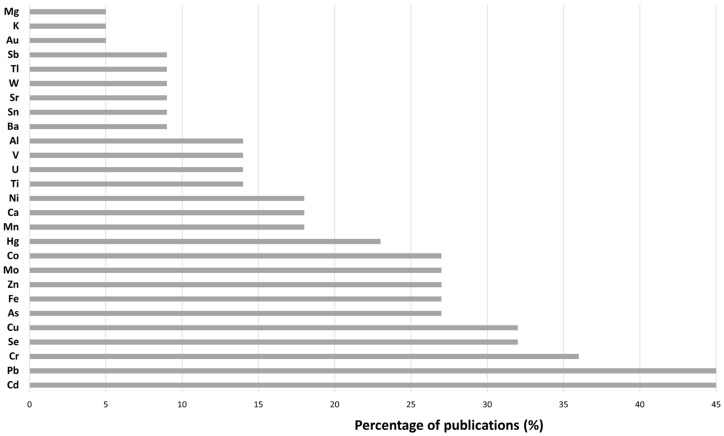
Detected and quantified analytes by ICP.

The most quantified analytes by ICP were Pb and Cd (45%), followed by Cr (36%), Cu (32%), Se (32%), and Zn (27%), as shown in Figure 9. It is worth mentioning that compared to AAS technique, a higher number of analytes was detected in seminal plasma using ICP. Specifically, the following analytes were detected by ICP but not by AAS: Al, Au, Ba, Mo, Ni, Sb, Sn, Sr, Ti, Tl, U, V, and W. This is due to the combination of lower LOD (see Figure 7) and the multi-elemental capability of ICP techniques. A table is vailable regarding information about the multi-elemental quantification of SP (Table 5).

The study groups of the articles using ICP were similar to those of the articles using AAS. So, we found groups of studies using fertile men as the control group and non-fertile men [30], and normal quality and low/abnormal seminal quality [44,45,81,83,85]. Further to this, some articles divided men into normozoospermic (N) men as the control group and oligospermic (O) men and azospermic (Azo) men [82], or oligoasthenozoospermic (OA) men [91]. Infertile men contained a higher content of Cd and Pb in their SP compared to fertile men [30]. However, while three articles showed that men with a lower/abnormal seminal quality had greater amounts of Pb and Cd in their SP [81,83,85], one article showed higher Pb SP levels in men with normal parameters and no difference in Cr levels in men that showed a pathological spermiogram [45].

Environmental exposure and its impact on seminal content was also tested. The “Land of Fires” region, which is in Southern Italy, is a highly environmentally polluted area with chemicals and heavy metals due to the uncontrolled spillage of industrial and urban waste and the burning of toxic waste [89]. Cr and Cu content in SP was investigated in a father and a son from the “Land of Fires”, which were higher compared to the control subject [89]. An excess of Cu and Cr was found in the father and son. Other elements such as Au and U have been quantified by ICP in SP. Au content was determined in men from an Au deposit area [34], while U was quantified in First Gulf War veterans. Two articles investigated the influence of implants on the content of Cr, Co, and Mo in SP [50,86].

#### 3.3.4. Total Reflection X-ray Fluorescence and Commercial Kits

A low number of articles used commercial kits (*n*: 4) and total reflection X-ray fluorescence (TXRF) (*n*: 2) for heavy metal quantification in SP. Table 8 summarizes the studies based on these methodologies. While commercial kits can be of interest, they do not have a multi-elemental capability. As regards TXRF, this technique has a multi-elemental capability, but LOD are too high to perform the trace analysis of some heavy metals [55].

##### Instrumental Settings for Heavy Metal Detection and Quantification

The equipment specifications of each tool used for heavy metal detection and quantification are available in Table 8. Values for LOD, LOQ, and recovery values were not detailed.

##### Heavy Metal Detection and Quantification

The results demonstrated that the most quantified analyte using a commercial kit was Zn [36,39,101], which was also quantified using TXRF [103,104] (Table 9). Other analytes such as Cu [103] and Se [104] were quantified in SP using TXRF.

Regarding the Zn levels found in seminal plasma samples, men with varicocele (Grades 2 and 3) displayed a lower content compared to normozoospermic men without this disease [103]. Further to this, one study [101] found that a group of men with varicocele of Grade 3 had the lowest Zn levels, followed by patients with Grades 1 and 2, while the control group had significantly higher values of Zn concentration [102]. Furthermore, Zn levels in SP were investigated in smokers and non-smoking patients [39]. Zn values were higher in the non-smoking group of men compared to the smokers. The decrease in concentration, motility, and morphology of sperm parameters was found to be significantly lower among smokers than non-smokers, and this decrease was found to be significantly greater in smokers with abnormal seminal plasma Zn levels when compared to smokers with normal seminal plasma Zn levels [39].

##### Sample Preparation

Firstly, semen samples are centrifugated to obtain SP [36,101,102]. Then, it can be frozen until heavy metal analysis [102]. Sample preparation can be made following the instructions of commercial kits. For TXRF, the sample can be diluted 1:1 with gallium as the internal standard [103] or 1:2 using high performance liquid chromatography water, (HPLC-H_2_O) and then gallium can be added [104].

## 4. Conclusions

Modern andrology requires new diagnostic options to complement well-established diagnostic techniques that can aid in understanding the underlying physiopathology of impaired fertility in men. In this context, the investigation of environmentally and/or occupationally exposed men to EDCs and their impact on male fertility represents an open window into the issue under study. Previous studies showed the negative influence of HMs on seminal parameters and molecular biomarkers such as ROS, DNA fragmentation, and epigenetic changes, altering sperm’s functionality. Fast, reliable, and sensitive quantification of EDCs in SP could be important for the development of accurate diagnostic and preventive strategies to address male infertility towards providing personalized therapy, as the interaction between genetic and environmental factors is key for screening the greatest informative biomarkers, assessing their potential, and validating new molecules as potential targets for personalized therapy [24].

In this context, this systematic review provides information about how to detect and quantify HMs in human seminal plasma by using analytical techniques from information about the sample preparation to its detection. HMs were measured in SP with AAS, ICP, TXRF, and commercial kits, and AAS and ICP were the most employed techniques. For the quantification of HMs, AAS was the most used technique and analytes such as Zn, Pb, and Cd were the major analytes quantified, while Hg and As were the minor ones. ICP was the second most employed technique, especially ICP-MS. A higher number of analytes was measured using this technique including Al, Au, Ba, Mo, Ni, Sb, Sn, Sr, Ti, Tl, U, V, and W, while the most quantified analytes were Pb, Cd, Cr and Cu. In contrast with this, TXRF and commercial kits were weakly used for HM detection and quantification with Zn being the most commonly measured analyte.

Regarding the analytical information provided by the articles included in this systematic review, in AAS, 37% showed values for LOD, while in ICP, 43% of the articles included data on LOD/LOQ. Related to sensitivity information, we found that only a few articles included this information, meaning that the information could not be reliable because recovery values were shown in only 9% of the articles for AAS and 24% in the articles using ICP. Considering the calibration techniques and quality controls used, 46% of the articles using AAS included this information versus 62% using ICP.

Although to the best of the authors’ knowledge, there is not yet any study demonstrating the capabilities of ICP-MS/MS for the multi-elemental trace analysis of SP samples, it is expected that, in the near future, this technique will be used for this purpose. This is because first, the limits of detection provided are extremely low; second, spectral interferences can be compensated for by using a collision–reaction cell as well as two quadrupole analyzers; and third, it is able to handle a salty matrix without any blocking problems.

## Figures and Tables

**Table 1:** Main country affiliation by first author for heavy metal research in SP.

Country	Number of Publications	Ref.
India	9	[19,27,28,29,30,31,32,33,34]
China	6	[35,36,37,38,39,40]
Poland	5	[41,42,43,44,45]
Egypt	5	[46,47,48,49,50]

**Table 2:** Chemical elements and their symbols cited during this review.

Symbol	Chemical Element	Symbol	Chemical Element
Au	Gold	Mo	Molybdenum
Al	Aluminum	Na	Sodium
As	Arsenic	Ni	Nickel
Ba	Barium	Pb	Lead
Ca	Calcium	Sb	Antimony
Cd	Cadmium	Se	Selenium
Co	Cobalt	Sn	Tin
Cr	Chromium	Sr	Strontium
Cu	Copper	Ti	Titanium
Fe	Iron	Tl	Thallium
Hg	Mercury	U	Uranium
K	Potassium	V	Vanadium
Mg	Magnesium	W	Wolframium
Mn	Manganese	Zn	Zinc

**Table 3:** Detected analytes, limits of detection (LOD), and AAS analytical tools used by articles.

Ref.	Detected Analyte	LOD	Analytical Tool
[19]	Cd and Pb	n.a	FAAS
[27]	Cd and As	As: 0.05 μg/L and Cd: 0.002 μg/L	AAS
[28]	Cu	n.a	AAS
[29]	Cd and Zn	Zn: 6.6 mg /L	FAAS
[32]	Fe	n.a	AAS
[35]	Cd, Zn, and Pb	n.a	AAS, GFAAS, and FAAS
[41]	Pb	n.a	GFAAS
[42]	Zn and Cu	n.a	AAS
[43]	Cd, Zn, Se, Pb, and Cu	n.a	ETA-AAS
[46]	Fe and Cd	n.a	AAS
[47]	Zn	n.a	FAAS
[48]	Zn	n.a	AAS
[49]	Pb	n.a	GFAAS
[56]	Fe, Zn, Cu, and Mg	n.a	AAS
[57]	Cd and Pb	Pb: 0.32 and Cd: 0.50 μg/L	GFAAS
[58]	Hg	0.1 μg/L	AAS
[59]	Cd, Zn, Se, and Pb	n.a	AAS
[60]	Zn	0.47 μg /L	GFAAS
[61]	Fe, Zn, Se, and Cu	n.a	FAAS and GFAAS
[62]	Pb	0.1 μg/L	AAS
[63]	Pb	0.32 μg/L	GFAAS
[64]	Hg	0.098 μg/L	AAS
[65]	Zn	5 μg/L	FAAS
[66]	Cd, Se, and Pb	n.a	AAS
[67]	Fe, Zn, Se, Cu, Ca, and Mg	n.a	FAAS
[68]	Zn, Mn, and Cu	n.a	AAS
[69]	Zn	n.a	AAS
[70]	Zn and Se	Zn: 0.47μg/L and Se: 0.78μg/L	AAS and FAAS
[71]	Zn	n.a	AAS
[72]	Cr and Co	0.02 mg/L	ETA-AAS
[73]	As, Cd, Cu, Pb, Se, and Zn	As: 2.59, Cd: 0.03, Cu:1.03, Pb: 0.98, Se: 0.02, and Zn: 0.065 μg/L	AAS, ETA-AAS, and HGAAS
[74]	Cd, Zn, Pb, Cr, and Cu	n.a	AAS
[75]	Cd and Pb	n.a	FAAS
[76]	Zn	n.a	AAS
[77]	Cd, Pb, and Cr	n.a	AAS

AAS: atomic absorption spectroscopy, ETA-AAS: electrothermal atomization - atomic absorption spectrometry, FAAS: Flame atomic absorption spectroscopy, GFAAS: graphite furnace atomic absorption spectrometry, HGAAS: Hydride generation atomic absorption spectroscopy, n.a: not available.

**Table 4:** Levels of Cd, Pb, and Zn measured by AAS. Concentrations are expressed in terms of median values or means ± SD.

Ref.	Study Group	Cd	Pb	Zn
[19]	Semen samples were collected from infertile male tea garden workers (*n*: 200) visiting fertility clinics in southern Assam, India. Age-matched donors as the control group (*n*: 200) who were not working in the tea gardens. All participants were residents of areas surrounding the tea gardens	Infertile: 0.0145 ppm	Infertile: 0.08 ppm	N.d
[27]	Proven fertile healthy volunteers (N, *n*: 32) and infertile individuals (*n*: 68): Oligo (*n*: 35) and Azo (*n*: 33) subjects, according to the WHO	N: 0.28, O: 0.41, Azo: 0.59 ppm	N.d	N.d
[28]	donors (*n*: 28) submitted semen samples for this study. They belonged to the province of Gujarat and were in the age group of 20–27 years. They were physically and mentally healthy	N.d	N.d	N.d
[29]	G1: fertile smokers (*n*: 68), G2: infertile non-smokers (history of genital examination (testis and scrotum), family inheritance) (*n:* 72), G3: infertile smokers (*n:* 76), and G4: non-smoker fertile subjects (*n:* 74), as the control group	G1: 6.8 ± 0.65, G2: 6.9 ± 1.77, G3: 12 ± 1.52, G4: 4.02 ± 0.62 μg/mL	N.d	G1: 0.71 ± 0.08, G2: 0.12 ± 0.03, G3: 0.07 ± 0.02, G4: 1.99 ± 0.09 mg/mL
[32]	N (*n*: 19), O (*n*: 11), Azo (*n*: 12), OA (*n*: 18), A (*n*: 15) according to the WHO	N.d	N.d	N.d
[35]	Subjects infected with HIV were recruited from Chongqing AIDS Hospice, Chongqing, China (*n*: 50)	1.69 ± 0.33 μg/L	8.57 ± 0.86 μg/L	30.66 ± 1.60 mg/L
[41]	Based on the medians of the levels of Zn in seminal plasma, the study subjects were divided into different groups: men with low environmental exposure to Zn (Zn-L) (*n:* 33) and men with high environmental exposure to Cu (Zn-H) (*n*: 32)	N.d	N.d	Zn-L: 85 ± 42.5, Zn-H: 55.0 ± 58.2 mg/dL
[42]	Based on the median of the values of Pb concentration in seminal plasma (PbS = 1.00 μg/dL), the subjects were divided into two groups: low environmental exposure to Pb (LE)–Pb concentration in seminal plasma between 0.40 and 1.00 μg/dL and a group with high environmental exposure to Pb (HE)–Pb concentration in seminal plasma between 1.01 and 2.70 μg/dL. N_T_: 65 study participants	N.d	LE: 0.72 ± 0.19, HE: 1.54 ± 0.47 μg/dL	N.d
[43]	Men were divided into two groups of proven fertile men (*n*: 85) and infertile patients (*n*: 131), who had been treated due to infertility for a period longer than 1 year. N_T_: 216, men	Proven fertility: 0.76, infertile patients: 0.92 μg/dL	Proven fertility: 0.76, infetile patients: 0.92 μg/dL	Proven fertility: 0.47, infetile patients: 0.42 μg/dL
[46]	Subjects (N_T_: 216), G1 (*n*: 70) N samples as the control, G2 (*n*: 48) A samples, G3 (*n*: 18) OA samples, G4 (*n*: 41) OAT samples, G5 (*n*: 39) Azo samples, according to the WHO	N: 0.015 ± 0.005, A: 0.065 ± 0.017, OA: 0.114 ± 0.018, OAT: 0.072 ± 0.013, Azo: 0.083 ± 0.028 μg/mg	N.d	N.d
[47]	Men (*n*: 160) recruited from the Andrology Unit, University Hospital. They were divided into healthy fertile non-smokers, G1 (*n*: 80), and fertile smokers, G2 (*n*: 80)	N.d	N.d	G1: 139.51 ± 7.78, G2: 101.21 ± 11.03 μg/mL
[48]	OAT non-smokers (*n:* 72), OAT smokers (*n*: 84), OAT non-smokers ith Vx (*n*: 23), and OAT smokers with Vx (*n*: 67)	N.d	N.d	OAT non-smoker: 97.2 ± 7.3, OAT smoker: 79.16 ± 5.6, OAT non-smoker with Vx: 68.2 ± 4.6, OAT smoker with Vx: 52.73 ± 9.96 μg /mL
[49]	Men with primary infertility (*n*: 29) attending the outpatient clinic of infertility in Mansoura University Hospital, Egypt	N.d	11.40 ± 7.53 μg/dL	N.d
[56]	Men referred to clinical laboratories in Gorgan (northern Iran) for routine semen analysis as the primary approach for male fertility testing. The control group included N subjects (*n*: 96) and the case group included A subjects (*n*: 96). N_T_: 192	N.d	N.d	N: 188.42 ± 99.61, A: 187.15 ± 87.38 mg/mL
[57]	Male partners (*n*: 300) of couples investigated for infertility. Males with known causes of infertility were excluded. Environmentally and occupationally exposed (*n*: 164) and non-exposed (*n*: 136) men	EE: 0.7, N-EE: 1.3; OE:1.4, N-OE: 1; E and/or OE: 1.2, N-EE and/or OE 1.1 μg/dL	EE: 19.7, N-EE: 14.3; OE: 15.9, N-OE: 15.7; E and/or OE: 17.7, N-EE and/or OE 13.5 μg/dL	x
[58]	Case subjects (*n*: 30) composed of men with OAT and control subjects (*n*: 31) composed of N patients, according to the WHO	N.d	N.d	N.d
[59]	Azo (*n*: 30), Oligo (*n*: 50), and N (*n*: 50) according to the WHO	N: 0.41 ± 0.15, O: 0.47 ± 0.16, Azo: 0.37 ± 0.13 μg/L	N: 0.47 ± 0.17, O: 0.53 ± 0.17, Azo: 0.43 ± 0.15 μg/L	N: 100.53 ± 16.85, O: 104.56 ± 15.94, Azo: 100.310 ± 3.14 μg/L
[60]	Male partners (*n*: 120) from couples in infertility treatment. G1 (*n*: 40) consisted of males with normal ejaculate (N), G2 (*n*: 45) consisted of A patients, and G3 which consisted of OAT	N.d	N.d	Control: 97.6 ± 34.9, A: 43.4 ± 24.9, OAT: 32.4 ± 7.9 mg/L
[61]	Semen samples were obtained from men aged between 24 and 38 years attending a fertility clinic. N (*n*: 6), T (*n*: 6), AT (*n:* 6), OT (*n*: 6), according to WHO guidelines	N.d	N.d	N: 150.67 ± 4.75, T: 127.00 ± 12.37, AT: 127.50 ± 5.30, OT: 120.40 ± 13.98 mg/L
[62]	Male partners of infertile couples attending the reproductive center of Lin-Kou Medical Center (*n:* 341), Chang Gung Memorial Hospital and undergoing infertility evaluation were recruited to the study	N.d	2.19 ± 1.45 μg/L	N.d
[63]	Male partners (*n:* 300) of couples investigated for infertility at Vindana Reproductive Health Center, Colombo, were recruited after informed consent was obtained. Normozoospermics (*n:* 77) and pathozoospermics (*n:* 38) following the WHO guidelines	N.d	Total: 15.7 μg/dL; P: 17.2 ± 3.02, N: 15.0 ± 1.70 μg/dL	N.d
[64]	Men (*n:* 179), men with normal sperm parameter (*n:* 48), and men with abnormal sperm parameter (*n:* 131)	N.d	N.d	N.d
[65]	Three male groups (age: 20–55 years) based on sperm count: O (*n:* 40); Azo (*n:* 20); and healthy fertile control males (*n:* 40)	N.d	N.d	Control: 29.85 ± 1.67, O: 32.77 ± 2.41, Azo: 38.20 ± 3.68 μmol/L
[66]	Infertile men (test group, *n:* 20) attending various fertility centers in Faisalabad, Pakistan were recruited in the study and fertile male volunteers (control group *n:* 20), according to the WHO	Fertile: 1.43 ± 0.85, infertile: 9.11± 2.34 μg/L	Fertile: 3.2 ± 1.43, infertile: 10.20 ± 4.54 μg/L	N.d
[67]	Two groups: a low level of metals group (Me-L, *n:* 44) and a high level of metals group (Me-H, *n:* 44)	N.d	N.d	ME-L: 119.29 ± 39.66, ME-H: 139.14 ± 70.45 mg/dL
[68]	Semen samples (*n:* 216) were divided into 5 groups based on WHO criteria. G1 (*n:* 70) N as the control and 4 groups of infertile males: G2 (*n:* 48) A samples, G3 (*n:* 18) OA, G4 (*n:* 41) OAT samples, and G5 (*n:* 39) Azo samples	N.d	N.d	G1: 3.93 ± 0.76, G2: 1.91 ± 0.56, G3: 1.1 ± 0.29, G4: 2.17 ± 0.56, G5: 2.13 ± 0.61 μg/mg
[69]	Patients with SCI (*n:* 24) were selected from the multidisciplinary clinic and controls (*n:* 24) were selected from the family planning clinic of the university hospital matched for age	N.d	N.d	SCI patients: 85.20 ± 65.52 and controls: 147.16 ± 72.17 mg/L
[70]	N (*n:* 60), A (*n:* 74), O (*n:* 56), T (*n:* 60) subjects according to the WHO	N.d	N.d	N: 144 ± 42.13, A: 122 ± 34.69, O: 120.51 ± 25.33, T: 126 ± 24.82 mg/L
[71]	Men (*n:* 276) diagnosed with O (*n:* 100) and N (*n:* 176) according to the 2010 WHO guidelines. Subjects diagnosed with low sperm count (*n:* 100, *n:* 26 were Azo)	N.d	N.d	Azo: 1.1, O: 1.75 mmol/L
[72]	Patients (*n:* 50) were enrolled for analysis. Patients in each group were based on the implant type: metal-on-metal (*n:* 25) and metal-on-polyethylene (*n:* 25) implants	N.d	N.d	N.d
[73]	Male human subjects from Taiwan (*n:* 196)	0.5 ± 0.3 μg/L	0.6 ± 1.1 μg/L	169.3 ± 97.9 μg/L
[74]	Semen samples were collected from clinically diagnosed O males (*n:* 120). Groups per age: 18–23 (*n:* 20), 24–29 (*n:* 20), 30–35 (*n:* 20), 36–41 (*n:* 20), 42–47 (*n:* 20), >47 (*n:* 20) and a control group of N men (*n:* 30)	18–23: 0.039, 24–29: 0.037, 30–35: 0.046, 36–41: 0.047, 42–47: 0.050, >47: 0.091, and control: 0.008 μg/mL	18–23: 0.028, 24–29: 0.117, 30–35: 0.229, 36–41: 0.110, 42–47: 0.231, >47: 0.222, and control: 0.010 μg/mL	18–23: 2.913, 24–29: 1.628, 30–35: 1.299, 36–41: 0.143, 42–47: 2.469, >47: 1.830, and control: 0.115 μg/mL
[75]	Males (*n:* 101) at Kamal-Alsamerae Hospital, including infertile patients (*n:* 70) with ages ranging (22–50) years compared to fertile males as the control (*n:* 40). N individuals (control group) (*n:* 37), infertile individuals (OA) (*n:* 32), and infertile individuals (Azo) (*n:* 32)	N: 0.141 ± 0.028, OA: 0.134 ± 0.020, Azo: 0.089 ± 0.032 μg/mL	N: 0.830 ± 0.213, OA: 0.693 ± 0.131, Azo: 0.045 ± 0.020 mg/mL	N.d
[76]	Couples (*n:* 253) were enrolled from an IVF center, Department of Obstetrics and Gynaecology, Institute of Kidney Diseases and Research Centre, Ahmedabad, India. ET conducted (*n:* 176) and ET not conducted (*n:* 77)	N.d	N.d	ET conducted: 78.63 ± 4.79, ET not conducted: 65.25 ± 5.22 mg/L
[77]	Males attending a fertility clinic (*n:* 78) at the University of Calabar Teaching Hospital with confirmed infertility were used as subjects. They were classified as O (*n:* 18), OA (*n:* 40) and Azo (*n:* 20). Fertile men with a history of fathering at least one child were used as the controls (*n:* 62)	Control: 3.66 ± 0.22, OA: 3.65± 0.23, O: 3.32 ± 0.4, Azo: 3.49 ± 0.53 μg/dL	Control: 17.13 ± 0.69, OA: 26.81 ± 7.55, O: 15.41 ± 1.48, Azo: 12.2 ± 0.82 μg/dL	N.d

A: asthenozoospermia, AT: asthenoteratozoospermia, Azo: azospermia, EE: environmentally exposed, ET: embryo transfer, E and/or OE: environmentally and/or occupationally exposed, N: normozoospermia, N.d: non-detected, N-EE: non-environmentally exposed, N-EE and/or OE: non-environmentally and/or occupationally exposed, N-OE: non-occupationally exposed, SCI: spinal cord injury, O: oligozospermia, OAT: oligoasthenozoospermia, OE: occupationally exposed, OT: oligoteratozoospermia, P: pathozoospermia, T: teratozoospermia, Vx: varicocele.

**Table 5:** Detected analytes, limits of detection (LOD) and quantification (LOQ), and analytical tools used by articles.

Ref.	Detected Analytes	LOD/LOQ	Analytical Tool
[30]	Cd and Pb	LOD were Pb:1.9 and Cd:0.28 μg/L	ICP-AES
[31]	Cd and Pb	LOD were Pb:1.9 and Cd:0.28 μg/L	ICP-AES
[34]	Au	n.a	ICP-AES
[37]	Cd, Se, Pb, As, Cr, and Ni	n.a	ICP-MS
[38]	Cd, Zn, Se, As, V, Cu, and Sn	LOQ for V was 0.0046 μg/L	ICP-MS
[40]	Cd, Zn, Se, As, Mn, Co, Cu, Mo, and Tl	n.a	ICP-MS
[44]	Fe and Zn	n.a	ICP-MS
[45]	Pb, Cr, and Co	n.a	ICP-MS
[50]	Cr, Co, and Mo	n.a	ICP-MS
[81]	Cd, Pb, Hg, As, Ba, and U	LOD Hg: 0.5 μg/L and 1 μg/L for all other metals	ICP-MS
[82]	Cd, Zn, Pb, Hg, Ba, Mg, Ca, Al, Ti, V, Cr, Mn, Co, Ni, Cu, Sr, Sn, Sb, and Mo	n.a	ICP MS
[83]	Cd, Se, Pb, Hg, As, Al, Ti, V, Cr, Mn, Ni, Cu, Sr, Mo, and W	LOD Zn and Al: 2.5 μg/L, and 0.5 μg/L for all other metals	ICP-MS
[84]	Fe, Cd, Se, Pb, As, U, Al, Cr, Mn, Co, Ni, Cu, Sb, Mo, W, and Tl	LOQ were Al: 0.25, Cr: 0.032, Mn: 0.060, Fe: 0.41, Co: 0.0040, Ni: 0.036, Cu: 0.13, Zn: 0.49, As: 0.014, Se: 0.047, Mo: 0.033, Cd: 0.0037, Sn: 0.020, Sb: 0.0030, W: 0.055, Tl: 0.0026, Pb: 0.0063, and U: 0.0020 μg/L	ICP-MS
[85]	Se, Pb, and Hg	LOD were Hg: 0.11, Pb: 0.74, and Se: 0.49 μg/L.	ICP-MS
[86]	Cr, Co, and Mo	LOD: 0.01 to 0.03 μg/L	ICP-MS
[87]	U	n.a	ICP-MS
[88]	Zn, Pb, and Hg	LOD were Hg: 0.1 μg/L, Cd: 0.1 μg/L, and Pb: 0.23 μg/L	ICP-MS
[89]	Cr and Cu	n.a	ICP-MS
[90]	Zn and Se	n.a	ICP-AES
[91]	Fe, Zn, and Ca	n.a	ICP-OES

ICP-AES: inductively coupled plasma atomic emission spectroscopy, ICP-MS: inductively coupled plasma mass spectrometry, ICP-OES: inductively coupled plasma optical emission spectroscopy, n.a: not available.

**Table 6:** ICP-MS spectroscopic interferences found when analyzing biological samples for nuclides of various elements.

Isotope	Abundance (%)	Interference from the Matrix/from the Argon Plasma
^24^Mg^+^	79.0	^12^C_2_^+^
^25^Mg^+^	10.0	^12^C_2_^1^H^+^, ^13^C^12^C^+^
^26^Mg^+^	11.0	^12^C^14^N^+^, ^12^C_2_^1^H_2_^+^, ^12^C^13^C^1^H^+^
^27^Al^+^	100	^12^C^15^N^+^, ^13^C^14^N^+^, ^1^H^12^C^14^N^+^
^47^Ti^+^	7.4	^12^C^35^Cl^+^
^48^Ti^+^	73.7	^12^C_4_^+^, ^36^Ar^12^C^+^
^49^Ti^+^	5.4	^36^Ar^13^C^+^, ^36^Ar^12^C^1^H^+^, ^12^C^37^Cl^+^
^52^Cr^+^	83.8	^40^Ar^12^C^+^, ^35^Cl^17^O^+^
^53^Cr^+^	9.5	^40^Ar^13^C^+^
^56^Fe^+^	91.7	^40^Ca^16^O^+^,^40^Ar^16^O^+^
^60^Ni^+^	26.2	^12^C^16^O_3_^+^
^63^Cu^+^	69.2	^36^Ar^12^C^14^N^1^H^+^, ^14^N^12^C^37^Cl^+^, ^16^O^12^C^35^Cl^+^
^65^Cu^+^	30.9	^12^C^16^O^37^Cl^+^, ^12^C^18^O^35^Cl^+^
^75^As^+^	100	^23^Na^12^C^40^Ar, ^12^C^31^P^16^O_2_^+^, ^40^Ar^35^Cl^+^, ^36^Ar^39^K^+^
^77^Se^+^	7.6	^12^C^19^F^14^N^16^O_2_^+^
^80^Se^+^	49.8	^40^Ar^40^Ca^+^, ^40^Ar^40^K^+^, ^40^Ar_2_
^112^Cd^+^	24.1	^40^Ca_2_^16^O_2_^+^

**Table 7:** Levels of Cd, Pb, and Cr measured by ICP. Concentrations are expressed in terms of median values or means ± SD.

Ref.	Study Groups	Cd	Pb	Cr
[30]	Fertile group (*n:* 46) (fertile men with proven fertility) and infertile men (*n:* 73)	Fertile: 4.03 ± 0.32 and infertile: 5.91 ± 0.25 μg/dL	Fertile: 5.30 ± 0.3, infertile: 7.24 ± 0.27 μg/dL	N.d
[31]	Male partners attending the Andrology Laboratory of the Reproductive Biology Department (*n:* 60) from the India Institute of Medical Sciences (AIIMS) in New Delhi, India	4.91 ± 2.12 μg/dL	6.18 ± 2.16 μg/dL	N.d
[34]	Subjects from a Au deposit area (*n:* 11) and from the control area (*n:* 13)	N.d	N.d	N.d
[37]	Men from couples (*n:* 103) undergoing their first IVF treatment in a reproductive center (Shenyang, China) were recruited	1.23 ± 0.85 μg/L	7.44 ± 44.61 μg/L	13.65 ± 17.53 μg/L
[38]	Eligible men (*n:* 746) with no self-reported diseases	0.37 μg/L	N.d	N.d
[40]	Subjects recruited (*n:* 1136). 464 men were tested for seminal androgen levels (testosterone, dihydrotestosterone (DHT), dehydroepiandrosterone (DHEA), and androstenedione (ADD)).	Total: 0.57, subjects with androgens: 0.53, without androgens: 0.60 μg/L	N.d	N.d
[44]	Samples from two groups: N men (*n:* 38) and men with abnormal seminal samples (*n:* 124)	N.d	N.d	N.d
[45]	Samples collected from G1 (N, *n:* 38) and G2 (pathological spermiogram, *n:* 124)	N.d	Normo: 0.2569 ± 0.4295, no normo: 0.1898 ± 0.27 (mg/kg of dry weight)	Normo: 0.0001, no normo: 0.0001 mg/kg
[50]	Subjects with IMN (*n:* 60) ranging in ages from 29 to 58 years and 30 age-matched healthy controls. G1, with no metal implant in their bodies. Two subgroups: individuals with IMN of less than 5 years (group 2A) (*n:* 30) and those with IMN of more than 5 years (group 2B) (*n:* 30)	N.d	N.d	G1: 42.55 ± 2.73, 2A: 47.46 ± 0.35, 2B: 85.42 ± 6.13 μg/L
[81]	Low (*n:* 61) and normal (control group, *n:* 55) quality semen groups, according to the WHO	Low-quality semen group: 6.22 and control: 3.67 μg/L	Low-quality semen group: 5.88 and control: 4.70 μg/L	N.d
[82]	N (*n:* 25), O (n: 25), and Azo (*n:* 25) according to the WHO	N: 1.6, O: 1.08, Azo: 4.99 μg/L.	N: 17.8, O: 11, Azo: 44.87 μg/L	N: 8.41, O: 11.44, Azo: 21.71 μg/L
[83]	Normal (*n:* 64) and abnormal (*n:* 30) patients were recruited for routine semen analysis in the Reproduction Biology Laboratory of the University Hospital of Marseille (France)	N: 0.2 ± 0.4, Abnormal: 1.01 ± 0.7 μg/L	N: 0.5 ± 1.1, Abnormal: 1.75 ± 0.9 μg/L	N: 26.45 ± 9.5, Abnormal: 28.14 ± 10.1 μg/L
[84]	Subjects (*n:* 764) from subfertile couples from Wuhan reproductive medicine center for examination with no prior knowledge of male factor infertility and a control group (N, *n:* 482)	Abnormal: 0.37, control: 0.37 μg/L	Abnormal: 0.28, normal: 0.24 μg/L	Abnormal: 0.42, normal:0.42 μg/L
[85]	Male participants (*n:* 84) were enrolled from the Center for Reproductive Medicine and Science, Taipei Medical University (Taiwan). Groups were the low-quality semen group (*n:* 27) and high-quality semen group (*n:* 57)	N.d	Total: 13.5 ± 10.1, low-quality sperm: 14.7 ± 14.2, high-quality sperm: 12.8 ± 6.99 μg/L	N.d
[86]	Patients with MM THA (mean time from implantation, 4.1 years, *n:* 11) and control patients of comparable ages with no metal implant in their body (*n:* 5)	N.d	N.d	Non-detailed
[87]	Samples were collected from a cohort of the First Gulf War veterans (*n:* 34)	N.d	N.d	N.d
[88]	Male partners (*n:* 36) recruited to the Study of Metals and Assisted Reproductive Technologies	0.25 μg/L	0.66 μg/L	N.d
[89]	Semen samples of the father (50 years old) and son (18 years old) came from the “Land of Fires” (Italy) obtained by the Medicina Futura Center (Acerra Province of Naples). Two age-matched men as the controls	N.d	N.d	Control: 10, son: 225, and father: 350 μg/L
[90]	Men with severe inflammation in semen (*n:* 29), men with severe inflammation in their EPS and/or post-M (*n:* 31), men with mild inflammation (*n:* 24), men with oligozoospermia with no inflammation (*n:* 32), and the control group (*n:* 27) (asymptomatic inflammation-free fertile men–partners of pregnant women)	N.d	N.d	N.d
[91]	SP samples of three different diagnostic groups (N, A, and OA) were analyzed (*n:* 21)	N.d	N.d	N.d

A: asthenozoospermia, Azo: azospermia, EPS: expressed prostatic secretion, IMN: intramedullary nailing, MM THA: metal-on-metal total hip arthroplasty, N: normozoospermia, N.d: non-detected, O: oligozospermia, OA: oligoasthenozoospermia, Post-M: post-prostate-massage urine, SP: seminal plasma.

**Table 8:** Information about the equipment specification and detected analytes by commercial kits and TXRF.

Ref.	Equipment Specification	Detected Analytes
[36]	Zn commercial kit from Nanjing Xindi Biological Pharmaceutical Engineering Co., Ltd. (Nanjing, China)	Zn
[101]	Zn concentration was determined according to the colour intensity of samples after reaction with Zn and 5-Br-3′-phosphoadenosine-5′-phosphosulfate (5-Br-PAPS) in an alkalic buffer	Zn
[102]	Commercial reagent Cobas kit (IRON2Iron Gen.2) and Cobas kit (CA2 Calcium Gen.2)	Fe, Ca
[39]	Seminal plasma Zn quantitative assay kit (Shenzhen HuaKang Co. Ltd., China)	Zn
[103]	Spectrometer S2 PICOFOX (Bruker AXS, Madison, WI, USA)	Zn and Cu
[104]	Spectrometer S2 PICOFOX (Bruker-nano, Berlin, Germany)	Zn and Se

**Table 9:** Study groups and sample preparations for heavy metal detection in SP.

Ref.	Study Groups	Sample Preparation	Zn Values (Means ± SD)
[36]	Subfertile men whose partners had not conceived within 12 months after stopping use of contraception attending an infertility clinic (Nanjing Jinling Hospital), (*n:* 1010)	After liquefaction, semen samples were centrifuged at 12,000× *g* for 5 min. The upper layer of SP was collected	9592.62 ± 1.16 mmol/L
[101]	Male subjects (*n:* 179) with a more than 1-year history of infertility and clinical Vx: Grade 1 (*n:* 9), Grade 2 (*n:* 36), and Grade 3 (*n:* 134). The control group included healthy subjects (*n:* 179)	To analyze Zn concentration in SP, sperm samples were centrifuged during 10 min at 1500× *g* to acquire the supernatant. Zn concentration was determined according to the colour intensity of samples after reaction with Zn and 5-Br-PAPS in an alkalic buffer	Vx Grade 1: 0.294 ± 0.045, Vx Grade 2: 0.240 ± 0.067, Vx Grade 3: 0.194 ± 0.085, and control subjects: 0.297 ± 0.045 μmol/mL
[102]	Three patient groups based on the ejaculate parameters: group T (*n:* 32), group AT (*n:* 27), and TL (*n:* 27). Healthy donors (with normal semen profiles and proven fertility) were considered the control group (*n:* 29)	After semen analysis, each aliquot of the fresh semen was centrifugated at 3500 rpm for 10 min to obtain the SP. It was frozen at −20 °C until trace element analysis	N.d
[39]	Male patients attending infertility investigation. Non-smoker (*n:* 79) and smoker (*n:* 68) patients	The SP was diluted in 1 mol/l ammonia/ammonium chloride buffer (1:150), pH 10.0, and estimated using a spectrophotometer	Non-smokers: 2.38 ± 1.04, smokers: 1.84 ± 0.85 mmol/L
[103]	Patients (*n:* 67) who had Vx Grade 2 (*n:* 42) and Grade 3 (*n:* 25). N men without varicocele were the control group (*n:* 44)	SP was mixed 1:1 with gallium as the internal standard. Mixed fluid (10 μL) was deposited on pure quartz-polished sample carriers and air dried in a laminar flow cabinet. Then, they were analyzed by TXRF	N (n: 44): 160.0 ± 99.4, Vx (Grade 2 and 3): 136.9 ± 76.4 mg/L
[104]	SP samples were obtained from men visiting the Department of Urology (University Hospital Charité) for infertility diagnosis (vasectomized men, *n:* 4) and healthy volunteers (*n:* 18)	Samples were diluted 1:2 using HPLC-H_2_O. Gallium was added as the internal standard	143.6 ± 95.6 mg/L

5-Br-PAPS: 5-Br-3′-phosphoadenosine-5′-phosphosulfate, AT: asthenoteratozoospermia, HPLC-H_2_O: high performance liquid chromatography water, N: normozoospermic, N.d: non-detected, SP: seminal plasma, T: teratozoospermia, TL: teratoleucozoospermia, TXRF: total reflection X-ray fluorescence, Vx: varicocele.

## Supplementary Materials

The following supporting information can be downloaded at: https://www.mdpi.com/article/10.3390/life13040925/s1, Table S1: Equipment specifications for heavy metal quantification by AAS. Table S2: Equipment specifications for heavy metal quantification by ICP.
