# Supplementary material for: Analytical Determination of Heavy Metals in Human Seminal Plasma—A Systematic Review
Source: Life (Basel). 2023 Mar 31;13(4):925. doi: 10.3390/life13040925 (PMC10145970; doi:10.3390/life13040925)
Supplement: Supplementary file 1 [file life-13-00925-s001.zip › life-2222118-supplementary.pdf]

Table S1. Equipment specifications for heavy metal quantification by AAS. n.a: not available.

Ref.	Equipment specifications
[19]	n.a
[27]	Perkin Elmer 3110, Model: Graphite Furnace Vario 6
[28]	Perkin Elmer, USA
[29]	Perklin Elmer precisely Analyst 200
[32]	Perkin and Elmer model 305
[35]	PerkinElmer AAnalyst400 (PerkinElmer, USA)
[41]	Unicam 929 and 939OZ Atomic Absorption Spectrometerswith GF90 and GF90Z
[42]	Unicam 929 and 939OZ Atomic Absorption Spectrometers with GF90 and GF90Z
[43]	n.a
[46]	n.a
[47]	Buck model 210 VGP (Buck Scien-tific, Inc., Norwalk, CT)
[48]	Buckmodel 210 VGP (Buck Scientific Inc., Norwalk, CT, USA)
[49]	Zeeman 5000 atomic absorption spectrophotometer, HGA 500; Perkin Elmer, Norwalk, CT, USA)
[56]	YOUNGLIN AAS 8020 (Hogye dong, Anyang, South Korea)
[57]	Varian spectra 250 (Australia)
[58]	DMA-80 Direct Mercury Analyzer (Milestone, Shelton CT, USA)
[59]	n.a
[60]	Zeenit 700-Analytik-Jena Flame and Graphite-Furnace AAS (Germany), equipped with deuterium and Zeeman background correction
[61]	Perkin Elmer Zeeman 5100PC (Perkin-Elmer GmbH, Rodgau, Germany) provided with an HGA 600 graphite furnace programmer
[62]	Varian spectraAA 200Z (USA)
[63]	Varian Spectra 250 (Australia)
[64]	AAnalyst800 (Perkin Elmer, LabX, Midland, Canada)
[65]	Buck Model 210-VGI (Bulls Scientific, East Norwalk, CT)
[66]	n.a
[67]	n.a
[68]	n.a
[69]	Analyst 100 apparatus (Perkin-Elmer Life and AnalyticalSciences, Shelton, CT, USA)
[70]	Zeenit 700-Analytik-Jena equipped with deuterium and Zeeman background correction (Germany)
[71]	Olympus AU 680 autoanalyzer (Beckman Coulter, Tokyo, Japan)
[72]	SpectrAA-200Z (Varian, Palo Alto, CA) with Zeeman's background correction and an L'vov platform
[73]	n.a
[74]	n.a
[75]	n.a
[76]	n.a
[77]	n.a

n.a: not avaibale

Table S2. Equipment **specifications** for heavy metal quantification by ICP. n.a: not available.

Ref	Equipment specifications
[30]	JY 2000
[31]	JY 2000
[34]	n.a
[37]	700X (Agilent Technologies, USA)
[38]	7700X (Agilent Technologies, USA)
[40]	7700 series (Agilent Technologies, USA
[44]	7500ce (Agilent Technologies, Santa Clara, CA, USA).
[45]	7500ce (Agilent Technologies, Santa Clara, CA, USA).
[50]	7700 series (Agilent Technologies, Santa Clara, CA, USA).
[81]	Agilent 7500ce (Agilent Technologies, Germany) equipped with a cell dynamic range (CDR).
[82]	7500cx (Agilent Technologies, Santa Clara, CA, USA).
[83]	X Series II (Thermo Electron, Les Ulis, France) employing a collision cell
[84]	Agilent 770 (Agilent Technologies, USA)
[85]	Thermo X Series II
[86]	SCIEX Elan 6100 DRC (PerkinElmer Instruments, Norwalk, Connecticut)
[87]	ELAN DRC, II (Perkin Elmer, Waltham, MA)
[88]	Element 2 sector field ICP-MS (Thermo Fisher Scientific, Bremen, Germany).
[89]	n.a
[90]	Varian Liberty II axial (Varian Inc. Scientific Instruments, Mulgrave, Australia)
[91]	Agilent 5100 (Agilent Technologies, Santa Clara, CA, USA).

n.a: not avaibale
